# Possible absence of critical thickness and size effect in ultrathin perovskite ferroelectric films

**DOI:** 10.1038/ncomms15549

**Published:** 2017-06-06

**Authors:** Peng Gao, Zhangyuan Zhang, Mingqiang Li, Ryo Ishikawa, Bin Feng, Heng-Jui Liu, Yen-Lin Huang, Naoya Shibata, Xiumei Ma, Shulin Chen, Jingmin Zhang, Kaihui Liu, En-Ge Wang, Dapeng Yu, Lei Liao, Ying-Hao Chu, Yuichi Ikuhara

**Affiliations:** 1 Electron Microscopy Laboratory, School of Physics, Peking University, Beijing 100871, China; 2 Collaborative Innovation Centre of Quantum Matter, Beijing 100871, China; 3 Centre for Nanochemistry, College of Chemistry and Molecular Engineering, Peking University, Beijing 100871, China; 4 Academy for Advanced Interdisciplinary Studies, Peking University, Beijing 100871, China; 5 Department of Physics and Key Laboratory of Artificial Micro- and Nano-structures of Ministry of Education, Wuhan University, Wuhan 430072, China; 6 Institute of Engineering Innovation, The University of Tokyo, Tokyo 113–8656, Japan; 7 Department of Materials Science and Engineering, National Chung Hsing University, Taichung, Taiwan 40227, China; 8 Department of Materials Science and Engineering, National Chiao Tung University, Hsinchu, Taiwan 30010, China; 9 International Centre for Quantum Materials, School of Physics, Peking University, Beijing 100871, China; 10 Department of Physics, South University of Science and Technology of China, Shenzhen 518055, China; 11 Institute of Physics, Academia Sinica, Taipei, Taiwan 105, China; 12 Nanostructures Research Laboratory, Japan Fine Ceramic Centre, Nagoya 456-8587, Japan; 13 WPI Advanced Institute for Materials Research, Tohoku University, Sendai 980-8577, Japan

## Abstract

Although the size effect in ferroelectric thin films has been known for long time, the underlying mechanism is not yet fully understood and whether or not there is a critical thickness below which the ferroelectricity vanishes is still under debate. Here, we directly measure the thickness-dependent polarization in ultrathin PbZr_0.2_Ti_0.8_O_3_ films via quantitative annular bright field imaging. We find that the polarization is significantly suppressed for films <10-unit cells thick (∼4 nm). However, approximately the polarization never vanishes. The residual polarization is ∼16 μCcm^−2^ (∼17%) at 1.5-unit cells (∼0.6 nm) thick film on bare SrTiO_3_ and ∼22 μCcm^−2^ at 2-unit cells thick film on SrTiO_3_ with SrRuO_3_ electrode. The residual polarization in these ultrathin films is mainly attributed to the robust covalent Pb–O bond. Our atomic study provides new insights into mechanistic understanding of nanoscale ferroelectricity and the size effects.

## Abstract (web summary)

Understanding ferroelectricity at reduced dimensions will be important for future sub-nanoscale devices based on ferroelectrics. Using high resolution electron microscopy; Gao *et al*., observe the existence of a measurable polarization at a thickness of just 1.5-unit cells

Stable ferroelectricity in ultrathin films enables diverse nanoelectronic functions including high storage capacity of non-volatile ferroelectric memories. However, the existence of critical thickness below which the ferroelectricity disappears had been predicted long time ago due to the intrinsic size or depolarizing field effects^1^,^2^,^3^,^4^. Experimentally observed critical thicknesses for epitaxial ferroelectric films usually are a few unit cells. For example, ferroelectricity in PbTiO_3_ (PTO) was observed in 10-unit cells thick film by scanning probe^5^ and 3-unit cells by synchrotron X-ray diffraction^6^. In BaTiO_3_ (BTO) films, the critical thickness has been reported to range from 7-unit cells^7^ to 4-unit cells^8^. For BiFeO_3_ (BFO), the ferroelectricity has been detected in 4 and 5-unit cells thick films^9^,^10^. Some of these values are in good agreement with the theoretical work, for example, the critical thickness has been calculated to be 3-unit cells for PTO (ref. 11) and 6-unit cells for BTO (ref. 12), while other calculations suggested that the ferroelectricity might even exist in a single-unit cell thick pervoskite film with proper electrodes^13^, indicating the possible absence of a critical thickness. Furthermore, although the ferroelectricity does not disappear in the ultrathin films, the magnitude of polarization should be progressively decreased^14^,^15^,^16^ as the critical thickness is approached.

Despite intensive studies^5^,^6^,^7^,^8^,^9^,^10^,^11^,^12^,^13^,^14^,^15^,^16^,^17^,^18^,^19^,^20^,^21^,^22^, the possible presence of a critical thickness in ferroelectric thin films is debateable and the underlying physics of size effect is not yet fully understood because competing effects such as intrinsic size effect^1^,^2^, surface properties^2^,^23^,^24^,^25^, substrate strain conditions^26^,^27^, interface chemistry and electrical boundary conditions^13^,^16^,^28^,^29^,^30^,^31^,^32^ contribute to the measured properties and thus complicate the behaviour. Early studies suggested that the intrinsic size effect existed in ferroelectrics and a critical volume (typically at hundreds of nanometres) was required to align the electric dipoles^1^,^2^, while the later developments showed that the depolarizing field, which is caused by the incomplete compensation of polarization bound charges at interfaces, mainly account for the size effect^3^,^4^ and therefore the value of critical size should be much smaller than previous thought (at a few nanometres or tens of nanometres). The depolarizing field increases as the film thickness decreases, leading to the instability of ferroelectricity in the ultrathin films and fine particles^2^. Besides, the effects of interface and surface also become more pronounced when the film becomes thinner. For example, atomic displacements (ionic relaxation) in a conducting electrode^28^ or insulating substrate^29^, and strength of bonding^32^ have been found to be crucial for stabilizing the ferroelectricity in ultrathin films. The polarization of film could also be controlled by the vapour environments through ionic absorbate at the free surface^33^, while the polarization underneath, on the other hand, in turn, strongly influence the surface structure^25^.

It is therefore crucial to quantitatively determine the individual effects of the substrate, surface, intrinsic size and their interactions to explore the nanoscale ferroelectricity in thin films. In fact, the recent advancements of aberration corrected (scanning) transmission electron microscopy [(S)TEM] have made it possible to measure the atomic displacements at the interface and surface in ferroelectric thin films^31^. For example, Chisholm *et al*.^30^ observed that the ferroelectric displacements even occurred in the electrode SrRuO_3_ (SRO) layer adjacent to the interface. Particularly, the atomically resolved annular bright-field (ABF) imaging^34^ allows us to precisely quantify the localized polarization^25^ via robustly determining both cation and oxygen positions over a wide range of thickness. In contrast to the traditional electrical measurements that are not reliable for ultrathin films due to the leakage current issue^5^,^15^,^16^ or other indirect methods such as piezoelectric response^10^ and X-ray structural measurements^6^ which gather the response from the entire heterostructure (that is, surface, film and interface), the electron microscopy method enables us to precisely profile the structural parameters as well as the polarization throughout the thin film thus to isolate the roles from the surface^2^,^23^,^24^,^25^, electrode-ferroelectric interface^13^,^16^,^28^,^29^,^30^,^31^,^32^, substrate strain^26^,^27^ and other factors^2^,^3^,^4^. Nevertheless, systematic atomic-scale electron microscopy studies of thickness-dependent polarization in ultrathin ferroelectric films have rarely been reported mainly due to the difficulties in high quality ultrathin film growth and TEM specimen preparation, and quantitative imaging and analysis.

Here, we study the thickness-dependent polarization in PbZr_0.2_Ti_0.8_O_3_ (PZT) films down to a single-unit cell thick via quantitative ABF imaging in aberration corrected STEM. We find that the polarization (∼96 μCcm^−2^) in PZT on bare STO remains stable until the thickness becomes less than ∼10-unit cells (∼4 nm), below which the polarization gradually decreases as the thickness decreases, for example, ∼81 μCcm^−2^ for 8.5-unit cells, ∼54 μCcm^−2^ for 6.5-unit cells and ∼22 μCcm^−2^ for 4-unit cells. Despite significant suppression, the polarization does not disappear even at 1.5-unit cells (∼16 μCcm^−2^), indicating the absence of critical thickness in this perovskite film. For a comparison, we also study the PZT thin films on STO substrate with SRO bottom electrode, in which the polarization is reduced to ∼22 μCcm^−2^ for 2-unit cells. The atomic structure suggests that the residual polarization in the films below 3-unit cells is mainly attributed to the robust covalent Pb–O bond. These results provide new insights into mechanistic understanding of the nanoscale polarization and size effect in ferroelectric thin films.

## Results

### PZT films on bare STO

**Figure 1:**
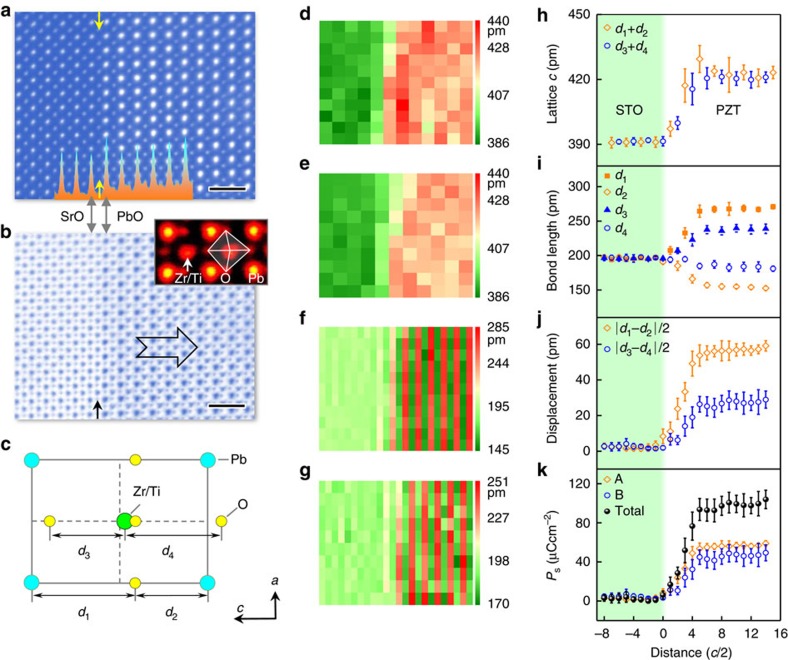
Quantitative measurements of polarization in PZT thin film on SrTiO_3_ substrate. This region is 24-unit cells thick. (**a**) A high angle annular field image of a substrate–film interface. The arrow shows the position of interface, TiO_2_ plane, between the first SrO and PbO planes. Inset: the interface is determined by the intensity profile. Scale bar, 1 nm. (**b**) The simultaneous recorded ABF image of the interface. Inset: enlarged view of PZT. The contrast is inverted for clarity. The polarization is upward, that is, pointing to the surface as denoted by the big arrow. Scale bar, 1 nm. (**c**) Schematic shows the projection of tetragonal PZT seen along [010] direction. *d*_1_ and *d*_2_ denote the long and short distances between Pb atoms (cyan) and O atoms (yellow) along the *c* direction; *d*_3_ and *d*_4_ denote the short and long distances between Zr/Ti atoms (green) and O atoms (yellow) along the *c* direction. Both SrTiO_3_ (STO) and PZT are expressed as ABO_3_ structure, where A represents for Sr and Pb, and B represents for Ti or Zr/Ti. The lattice constants *c* is calculated from the (**d**) A sublattice, and (**e**) B sublattice. (**f**) The calculated bond lengths *d*_1_ and *d*_2_ in the AO plane. (**g**) The calculated bond lengths *d*_3_ and *d*_4_ in the BO_2_ plane. (**h**) Mean of lattice *c*. The error bar is the s.d. (**i**) Mean of bond lengths of *d*_1_, *d*_2_, *d*_3_ and *d*_4_. The error bar is the s.d. (**j**) Averaged displacements of |*d*_1_*–d*_2_|/2 and |*d*_3_*–d*_4_|/2. The error bar is the s.d. (**k**) Mean of polarization calculated from the displacements in PbO plane, TiO_2_ plane and the total. The error bar is the s.d.

Figure 1 shows the interface of a PZT thin film with thickness ∼28 nm on a STO substrate viewing along [010]. Judged by the contrast in the high angle annular dark field image in Fig. 1a, the TiO_2_ plane between the SrO and PbO planes is labelled as the interface^29^. Figure 1b is the simultaneously recorded ABF image, from which both the cation columns (Pb and Zr/Ti) and oxygen columns are visible. The polarization in this sample is mainly upward^30^, that is, pointing towards right, because the O shifts to the left Pb column in Fig. 1b (ref. 25). The spontaneous polarization in each unit cell can be precisely calculated from: 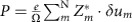, where *e* is the elementary charge, *Ω* is the volume of the unit cell, 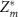 is Born effective charge^35^ and 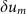 is the first-order change of the position vector of the *m*th basis atom (in Fig. 1c). The maps in Fig. 1d–g are the measured lattice constants and bond lengths. These values are averaged along the interface and plotted in Fig. 1h–j. A transition zone of ∼3-unit cells with suppressed polarization exists near the interface in the PZT film (so-called interfacial dead layer). Although the atomic displacements of Pb respective to O is much larger than that of Ti respective to O (ref. 36) in Fig. 1i,j, the Born effective charge for Pb is smaller^35^. As a result, the polarization contributed from PbO plane is close to that from TiO_2_ plane, as shown in Fig. 1k. The total spontaneous polarization in PZT film is measured to be ∼96 μCcm^−2^ (see details in the ‘Methods' section) and this measured value is representative (see Supplementary Fig. 1).

**Figure 2:**
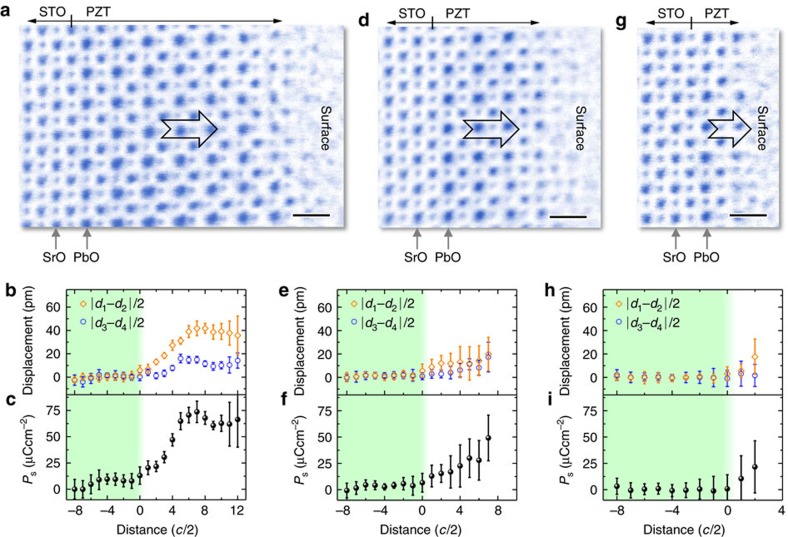
Thickness-dependent atomic displacements in PZT/SrTiO_3_. (**a**) An ABF image from a region with thickness of 6.5-unit cells. Scale bar, 0.5 nm. (**b**) Mean of displacements are calculated from of |*d*_1_*–d*_2_|/2 and |*d*_3_*–d*_4_|/2. The error bar is the s.d. (**c**) Mean of polarization calculated from the displacements. The error bar is the s.d. (**d**) An ABF image from a region with thickness of 4-unit cells. Scale bar, 0.5 nm. (**e**) Mean of displacements are calculated from of |*d*_1_*-d*_2_|/2 and |*d*_3_*-d*_4_|/2. The error bar is the s.d. (**f**) Mean of polarization calculated from the displacements. The error bar is the s.d. (**g**) An ABF image from a region with thickness of 1.5-unit cells. Scale bar, 0.5 nm. (**h**) Mean of displacements are calculated from of |*d*_1_*-d*_2_|/2 and |*d*_3_*-d*_4_|/2. The error bar is the s.d. (**i**) Mean of polarization calculated from the displacements. The error bar is the s.d.

A few regions with various thicknesses are investigated (see details in the ‘Methods' section). In a region with 8.5-unit cells thick in Supplementary Fig. 2, no significant difference than the bulk-like region is observed. At 6.5-unit cells in Fig. 2a–c (see also Supplementary Fig. 3), the displacements of Pb and Zr/Ti respective to O are reduced to ∼43 pm and ∼19 pm respectively, compared to ∼57 pm and ∼28 pm in Fig. 1, indicating the suppression of polarization. A dead layer ∼3-unit cells still exists at the substrate–film interface. The width of interfacial dead layer is almost independent of the film thickness. However, no such transition zone with suppressed polarization is observed at the surface in this upward domain, which is in good agreement with previous observations^25^. Further reducing the thickness to 4-unit cells in Fig. 2d–f (see also Supplementary Fig. 4), 2.5-unit cells in Supplementary Fig. 5, and 1.5-unit cells in Fig. 2g–i (see also Supplementary Fig. 6), the atomic displacements and polarization gradually decrease. However, they do not disappear even at 1.5-unit cells thick film in Fig. 2i, implying the polarization is suppressed but remains stable.

### PZT films on STO with SRO electrode

**Figure 3:**
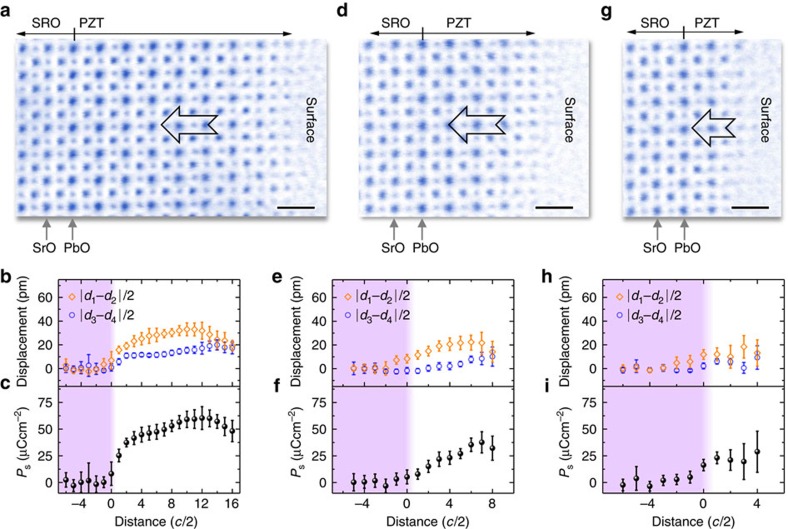
Thickness-dependent atomic displacements in PZT/SrRuO_3_/SrTiO_3_. (**a**) An ABF image from a region with thickness of 8-unit cells. Scale bar, 0.5 nm. (**b**) Mean of displacements are calculated from of |*d*_1_*–d*_2_|/2 and |*d*_3_*–d*_4_|/2. The error bar is the s.d. (**c**) Mean of polarization calculated from the displacements. The error bar is the s.d. (**d**) An ABF image from a region with thickness of 4-unit cells. Scale bar, 0.5 nm. (**e**) Mean of displacements are calculated from of |*d*_1_*–d*_2_|/2 and |*d*_3_*–d*_4_|/2. The error bar is the s.d. (**f**) Mean of polarization calculated from the displacements. The error bar is the s.d. (**g**) An ABF image from a region with thickness of 2-unit cells. Scale bar, 0.5 nm. (**h**) Mean of displacements are calculated from of |*d*_1_*–d*_2_|/2 and |*d*_3_*–d*_4_|/2. The error bar is the s.d. (**i**) Mean of polarization calculated from the displacements. The error bar is the s.d.

We also performed similar analysis for ultrathin PZT film on STO substrate with SRO bottom electrode in Supplementary Fig. 7 to study the behaviour of thickness-dependent polarization. The ABF images and measured data from three thicknesses (8, 4 and 2-unit cells) are shown in Fig. 3. Unlike the PZT film on bare STO with upward polarization, the polarization in PZT/SRO/STO is downward, pointing to the bottom interface, which is in good agreement with previous reports^30^,^31^. Besides the suppression of polarization at the bottom interface, the polarization also decreases at the top surface, which is consistent with previous studies of negatively poled PZT surface^25^,^31^. Similarly, in the thinner regions with thickness of 4-unit cells in Fig. 3d and 2-unit cells in Fig. 3g, the displacements (Fig. 3e,h) and the polarization (Fig. 3f,i) are also reduced. Still, they never disappear.

## Discussion

**Figure 4:**
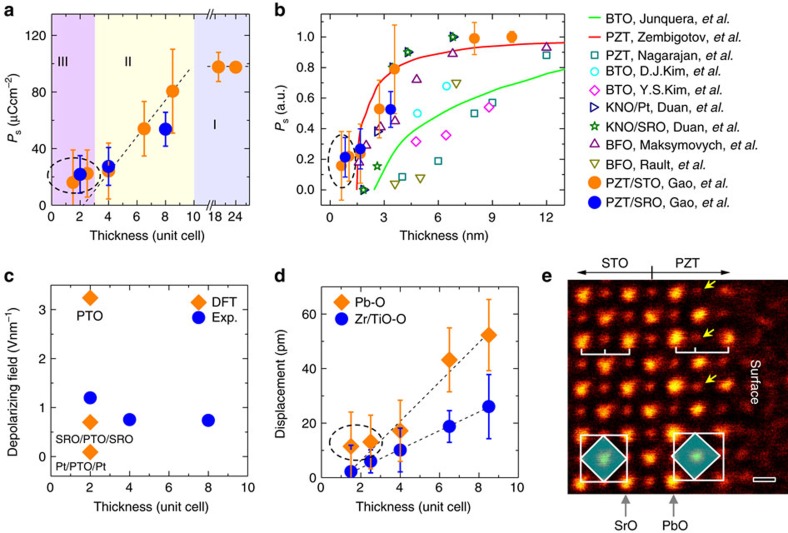
Thickness-dependent polarization. (**a**) Calculated polarization is plotted as a function of the thickness. The error bar is the s.d. The dashed lines are plotted for eye guidance. (**b**) Comparison of the normalized polarization as a function of thickness with previous results. Solid red plot shows the predictions from mean-field Landau-type model for PZT from ref. 26. The solid green plot is first principle calculations for SrRuO_3_/BaTiO_3_/SrRuO_3_ (SRO/BTO/SRO) from ref. 12. The blue squares are experimentally measured values for SRO/PZT/SRO from ref. 16. The cyan circles are experimentally measured values for SRO/BTO/SRO from ref. 22. The magenta diamonds are experimentally measured values for SRO/BTO/SRO from ref. 15. The orange triangles are theoretical predictions for Pt/KNbO_3_/Pt (Pt/KNO/Pt) from ref. 32. The dark green stars are theoretical predictions for SRO/KNO/SRO from ref. 32. The purple triangles are experimentally measured values for BiFeO_3_/La_0.67_Sr_0.33_MnO_3_/SrTiO_3_ (BFO/LSMO/STO) from ref. 10. The dark yellow triangles are experimentally measured values for BFO/LSMO/STO from ref. 21. The orange and blue solid discs are from this work. (**c**) Calculated depolarizing field is plotted as a function of the thickness for PZT on SRO/STO. The density function theory results from ref. 13 showing the depolarizing fields for free standing 2-unit cells thick PbTiO_3_ (PTO), 2-unit cells thick PTO sandwiched between SRO electrodes, and 2-unit cells thick PTO sandwiched between Pt electrodes. (**d**) Displacements of Pb respective to O and Zr/Ti respective to O are plotted as a function of the thickness for PZT on STO. The error bars are the s.d. (**e**) An ABF image of 1.5-unit cells thick PZT on STO. The contrast has been inverted for clarity. The octahedron shifting to left in the PZT film is visible by the naked eye. The arrows showing the O are closer to the left Pb, causing the atomic displacements and polarization. Scale bar, 0.2 nm.

The thicknesses-dependent polarization is summarized in Fig. 4a. Approximately, there are three regions: stable polarization zone I with film thickness ≥10-unit cells, progressive decayed polarization zone II between 10 and 3-unit cells, and weak polarization zone III with film thickness ≤3-unit cells. The polarization is upward in PZT on STO but downward with the SRO bottom electrode. As the thickness decreases, the magnitude of polarization is reduced. Interestingly, the orientation remains unchanged and no domain walls are observed in the ultrathin regions. The behaviour of thickness-dependent polarization magnitude in PZT films on both insulating STO and conducting SRO/STO is similar. In Fig. 4b, the measured polarization is normalized and compared with previous experimental and theoretical results. The presence of stable polarization in these ultrathin PZT films indicates that the polarization charges must be effectively compensated. The polarization screening can be from the external and/or internal charges^37^. The external screening is the charge compensation from the outside boundaries of ferroelectrics including both of top surface and bottom interface (that is, PZT/STO or PZT/SRO). The internal screening is determined by the distribution of free carriers or ionic charges and the associated structure changes within materials.

A rich variety of structures and properties were reported at the ferroelectric surface (refs 25, 33 and references therein). Although all the surfaces in these TEM specimens unavoidably contain thin amorphous layers that were introduced during the TEM specimen preparation, the surface structure is dominated by the polarization underneath while less affected by the thin-surface amorphous layer^25^. In the case of upward polarization in the PZT/STO, no significant surface reconstruction layer except the topmost atomic layer is observed for all the regions with different thickness in Fig. 2. This is in good agreement with previous experimental observations^25^. For the negatively poled surface in the PZT on SRO/STO, there is a thin reconstructed surface layer with reduced polarization, which was also reported before^31^. Nevertheless, despite different internal structure relaxation in the vicinity of top surfaces between PZT/STO and PZT/SRO/STO TEM samples, the external boundary conditions at the top surfaces should be similar, as they were prepared with the same procedures and in the same environment.

Therefore, the polarization directions (that is, upward for PZT on STO in ref. 30 and reference therein, and downward for PZT on SRO/STO in refs 30, 31) in these thin films should be governed by the boundary conditions of substrate/electrode. The width of interfacial deadlayer in PZT on SRO/STO is thinner than that in PZT/STO because the metallic SRO can provide negative charges to effectively screen the downward polarization at the interface and thus no significant internal structural relaxation within PZT is needed. For PZT on insulating STO substrate, formation of stripe domains^6^ or the atomic displacements in the insulating substrate^29^ were proposed to retain the stable polarization in ultrathin films. However, neither significant atomic displacements in the STO substrate nor 180° stripe domains in PZT is observed in our sample (that is consistent with previous study^30^ and references therein), suggesting either positive charges provided by STO or the internal charges within the PZT thin film at the interface mainly act as the screening charge^19^.

For the PZT film on STO with a bottom electrode SRO, the depolarizing field can be estimated from the short-circuit conditions *E*_d_=−2*λ*_eff_*P*(*dɛ*_0_)^−1^ (refs 14, 28, 38), where *E*_d_ is the depolarizing field, and *λ*_eff_ is the effective screening length, *ɛ*_0_is the permittivity of the free space, and *d* is the thin film thickness. Given a constant *λ*_eff_=0.02 nm (refs 12, 14, 38, 39 and references therein), the depolarizing field is approximately 0.7–1.2 Vnm^−1^ (in Fig. 4c) for PZT/SRO/STO heterostructure. The estimated values are in good agreement with the density function theory calculations^13^ which showed depolarizing field of 0.7 Vnm^−1^ for 2-unit cells thick PTO film sandwiched between SRO electrodes. For the PZT film on bare STO, calculation of the depolarizing field is usually based on open-circuit conditions, under which both of the polarization and depolarizing field strongly depend on the concentration of internal charges^40^. In this case, the depolarizing field mainly occurs at the bottom interface and top surface while almost vanishes in the middle of film^40^.

When the thickness becomes as thin as 3-unit cells, the bottom interface zone (that is, the area subjected to the substrate–film interface effects) and the surface zone (that is, the area subjected to the surface effects) become overlapped and interact with each other and thus complicate the screening mechanism. In fact, X-ray diffraction experiment of stripe domain patterns^6^ at room temperature and theories of effective Hamiltonian^11^ and *ab initio* calculation^24^ for *T*=0 K suggested that 3-unit cells is the critical thickness to retain the stable ferroelectricity in PTO films. In our study, when the thickness is <3-unit cells, the similar behaviour of suppressed polarization for both of the ultrathin PZT films on insulating substrate and metallic bottom electrode indicates that the compensation of polarization may be governed by the internal screening. Note both of the interface and surface contain ionic charges^25^,^30^,^33^,^39^ such as oxygen vacancies, Pb vacancies and absorbates. These defects may be even more easily introduced at the surface of TEM specimen foils during specimen preparation such as ion milling. In fact, the first principle calculations showed that the internal oxygen vacancies at the interface could effectively stabilize the polarization in ultrathin films^30^. Therefore, the small polarization can still survive because of the effective screening by these charged defects. Given polarization ∼16 μCcm^−2^ for 1.5-unit cells thick PZT film on STO substrate, it requires 8 at.% oxygen vacancy at the interface, that is, (PbSr)(ZrTi)O_2.92_, if the polarization is fully compensated by the interfacial oxygen vacancies. For the PZT film on SRO/STO, the polarization screening may come from both of the ionic charges and bottom electrode compensation.

In Fig. 1k, we show that the total polarization is attributed to the atomic displacements in both PbO and Zr/TiO_2_ planes. The displacements between Zr/Ti and O approximately linearly decrease and almost disappear below 3-unit cells for PZT on STO in Fig. 4d. However, the Pb–O displacements deviate from the linear decrease, instead they are even visible by naked eyes in the 1.5-unit cells region in Fig. 4e, where the arrows denote those oxygen columns shifting to the left Pb columns, similar to that in Fig. 1b. In the lead based ferroelectrics, the Pb–O bonds through the hybridization of Pb 6s and O 2p orbitals is covalent rather than pure ionic, and such covalent interaction accounts for the strong ferroelectric distortions^36^. Therefore, the residual polarization in the film below 3-unit cells is mainly contributed by the atomic displacements within the PbO planes due to the robust covalent Pb–O bond, while the Zr/TiO_2_ planes almost lose the polarization. Similar result for PZT on SRO/STO is shown in Supplementary Fig. 8.

In summary, we report a quantitative measurement of thickness-dependent polarization down to single-unit cell thick PZT films on STO substrate with and without bottom electrode. For the thick film (>10-unit cells) on bare STO, the polarization is mainly upward. The suppression of polarization only occurs at the substrate–ferroelectric interface to form a ferroelectric dead layer with thickness ∼3-unit cells, while at the top surface no significant surface structure except the topmost layer is observed. Between 10 and 3-unit cells, the polarization throughout the film is significantly suppressed, dominated by the thickness-dependent depolarizing field. Below 3-unit cells, the polarization is reduced but remains stable even for the PZT film on bare STO, for example, ∼22 μCcm^−2^ for 2.5-unit cells and ∼16 μCcm^−2^ (∼17%) for 1.5-unit cells. To best of our knowledge, the 1.5-unit cells thick PZT film is the thinnest perovskite film to experimentally show polarization so far. The observed polarization (or dipole moments) is stabilized by the soft phonon mode in ferroelectrics, which theoretically has a long wavelength. The analysed individual atomic terrace that is typically from 10 to 30-unit cells in width (see also Supplementary Fig. 9) is likely enough to stabilize the dipole moments at these regions^41^. However, the effects of the thicker regions adjacent to the thinner region cannot be completely excluded. Further studies are needed to address such effect in future. With the bottom electrode SRO, the polarization of PZT film is mainly downward. The suppression of polarization occurs both at the ferroelectric–electrode interface and top surface. At 2-unit cells, the average polarization is reduced to ∼22 μCcm^−2^. The atomic structure shows that the robust covalent Pb–O bonds mainly account for the residual polarization below 3-unit cells in the PZT films. Our study suggests that in practical thin films and devices the absence of critical thickness is possible due to the structural imperfection at the interface and surface.

## Methods

### Materials and characterization

Tetragonal PZT films oriented in the [001] direction were grown on (001) STO substrates with and without SRO bottom electrode by the pulsed laser deposition method using a KrF excimer laser (*λ*=248 nm). The laser beam was focused on the targets with an energy density of ∼2.5 Jcm^–2^ and repetition rate of 10 Hz. To grow the PZT thin film, the target with 5% excess lead was used to avoid the formation of lead deficiency in the film during the high temperature deposition. Growth was carried out at 650 °C and at oxygen pressure of 100 mTorr. In addition, for the sample with SRO bottom electrode, SRO was firstly deposited at 700 °C and 100 mTorr for obtaining higher quality thin film, and then cooled down to 650 °C with the same oxygen atmosphere for subsequent growth of PZT thin film. All samples were finally postannealed at the same temperature and at oxygen pressures of ∼300 Torr for 30 min, and then cooled down to room temperature with a ramp rate of 5 °C min^–1^. The sample with SRO bottom electrode has been checked by piezoresponse force microscopy, which is switchable and consistent with the previous results^16^. For the PZT on the insulating STO substrate, a 180° domain wall was observed in Supplementary Fig. 10, indicating the switchable nature. The mismatch between PZT and STO substrate is ∼1.2%. Such compressive strain favours the out-of-plane domain orientations. The thickness of as-grown PZT film is ∼28 nm. Cross-sectional TEM specimens were thinned to less than 30 μm by mechanical polishing and followed by argon ion milling in a Precision Ion Polishing System 691 (Gatan). Ion milling procedure consists of two steps. In the first stage of coarse milling, the guns were at 4 keV with angles 5° and −5°. In the following condition, the guns were set at 1 keV for 5 min with angles of 3.5° and −3.5°, and further lowered to 0.1 keV for 2 min for final surface cleaning. At the edges of the milled holes of the TEM specimen foils, the film thickness gradually changes from the full thickness to 0 nm, forming a series of flat atomic terraces with typical width between 10 and 30-unit cells (see Supplementary Fig. 9). To study the thickness-dependent polarization, STEM images were acquired from different regions with different thicknesses. ABF images were recorded at 300 kV in JEM ARM300CF (JEOL Ltd.). The convergence semi-angle for imaging is 24 mrad, collection semi-angles snap is 12−24 mrad for ABF imaging. All high-resolution ABF images used in this work were raw data without any post filtering. We deliberately choose those wider flat terraces and the middle of a single flat terrace for calculation to minimize the effects of the inhomogeneous thickness. Thus, the continuously changed thickness is not the main source of error in our case. The error bars of data points in Figs 2 and 3 mainly come from the (relatively random) spatial fluctuation of the structure in the TEM specimens.

### STEM image analysis

Atom positions were determined by simultaneously fitting with two-dimensional Gaussian peaks to an *a priori* perovskite unit cell using a MatLab code. The TiO_2_ plane between the first SrO and PbO planes is defined as the interface and labelled as the #0 atomic layer. To define the surface layer #*N* for a certain region, all the atom columns in the #*N* layer should be visible and thus can be fitted with Gaussian peaks for distance calculation, and then *N* is defined to be the thickness of the film. The lattice constant and bond-length for atomic columns in the #*n* layer are the distance between the #*n* and #(*n+1*) layer, where *n*≥2. Taking the oxygen sublattice as the reference (that is, 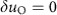), the displacements of Pb columns respective to the neighbouring O is 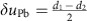 and Zr/Ti respective to the neighbouring O is 
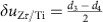
, where *d*_1_, *d*_2_ are long and short Pb–O bond length along *c* direction respectively, and *d*_3_, *d*_4_ are short and long Zr/Ti–O bond length along *c* direction respectively in Fig. 1c. The bond length *d*_*i*_ (*i*=1, 2, 3 and 4) are calculated on the basis of the fitted atomic positions. The values of Born effective charges for cubic STO and tetragonal PTO were calculated from *ab initio* theory^35^. Small displacements (a few picometres) can exist in the bulk substrate. Such small systematic-error-displacements and polarization originate from the tiny specimen mis-tilt (a few mrad) between optical axis and specimen^42^, which is unavoidable during experiments. These systematic-error-displacements are used as a reference for extracting the polarization in the ultrathin PZT films. The extracted polarization values in Figs 2 and 3 are calibrated by the references (that is, the mean of displacements in substrate/electrode bulk is set to be zero). The presence of point defects (for example, oxygen vacancies) would cause local structure distortion. Owing to the strong channelling effect along the atomic column, we can precisely determine the atomic column positions even including some point defects. For a specific atomic column, one oxygen vacancy may cause surrounding atom-position-shift. However, this shifted atom should not change the image contrast in the STEM image because the well-aligned atoms will dominate the total contrast due to the strong channelling effects. Therefore, the effects of point defects that cause localized structure distortion can be negligible in the STEM images.

### Data availability

The data that support the findings of this study are available from the corresponding author upon request.

## Additional information

**How to cite this article:** Gao, P. *et al*. Possible absence of critical thickness and size effect in ultrathin perovskite ferroelectric films. *Nat. Commun.* **8,** 15549 doi: 10.1038/ncomms15549 (2017).

**Publisher's note:** Springer Nature remains neutral with regard to jurisdictional claims in published maps and institutional affiliations.

## Supplementary Material

Supplementary Information Supplementary Figures and Supplementary Reference
