# Supplementary material for: Possible absence of critical thickness and size effect in ultrathin perovskite ferroelectric films
Source: Nat Commun. 2017 Jun 6;8:15549. doi: 10.1038/ncomms15549 (PMC5467161; doi:10.1038/ncomms15549)
Supplement: Supplementary Information — Supplementary Figures and Supplementary Reference [file ncomms15549-s1.pdf]

Supplementary Information for:

Possible Absence of Critical Thickness and Size Effect in Ultrathin Perovskite Ferroelectric Films

Peng Gao[1,2,3,4]*, Zhangyuan Zhang[1,5], Mingqiang Li[1,4], Ryo Ishikawa[6], Bin Feng[6], Heng-Jui Liu[7], Yen-Lin Huang[8], Naoya Shibata[6], Xiumei Ma[1], Shulin Chen[1], Jingmin Zhang[1], Kaihui Liu[2,3,4], En-Ge Wang[2,9], Dapeng Yu[1,2,10], Lei Liao[5]*, Ying-Hao Chu[8,11], Yuichi Ikuhara[6,12,13]*,

[1]*Electron Microscopy Laboratory, School of Physics, Peking University, Beijing 100871, China*
[2]*Collaborative Innovation Centre of Quantum Matter, Beijing 100871, China*
[3]*Centre for Nanochemistry, College of Chemistry and Molecular Engineering, Peking University, Beijing 100871, China*
[4]*Academy for Advanced Interdisciplinary Studies, Peking University, Beijing 100871, China.*
[5]*Department of Physics and Key Laboratory of Artificial Micro- and Nano-structures of Ministry of Education, Wuhan University, Wuhan 430072, China.*
[6]*Institute of Engineering Innovation, The University of Tokyo, Tokyo 113–8656, Japan.*
[7]*Department of Materials Science and Engineering, National Chung Hsing University, Taichung 40227, Taiwan, ROC.*
[8]*Department of Materials Science and Engineering, National Chiao Tung University, Hsinchu 30010, Taiwan, ROC.*
[9]*International Centre for Quantum Materials, Peking University, Beijing 100871, China.*
[10]*Department of Physics, South University of Science and Technology of China, Shenzhen 518055, China.*
[11]*Institute of Physics, Academia Sinica, Taipei 11529, Taiwan, ROC*
[12]*Nanostructures Research Laboratory, Japan Fine Ceramic Centre, Nagoya 456-8587, Japan*
[13]*WPI Advanced Institute for Materials Research, Tohoku University, Sendai 980-8577, Japan*

*e-mail: p-gao@pku.edu.cn; liaolei@whu.edu.cn; ikuhara@sigma.t.u-tokyo.ac.jp.

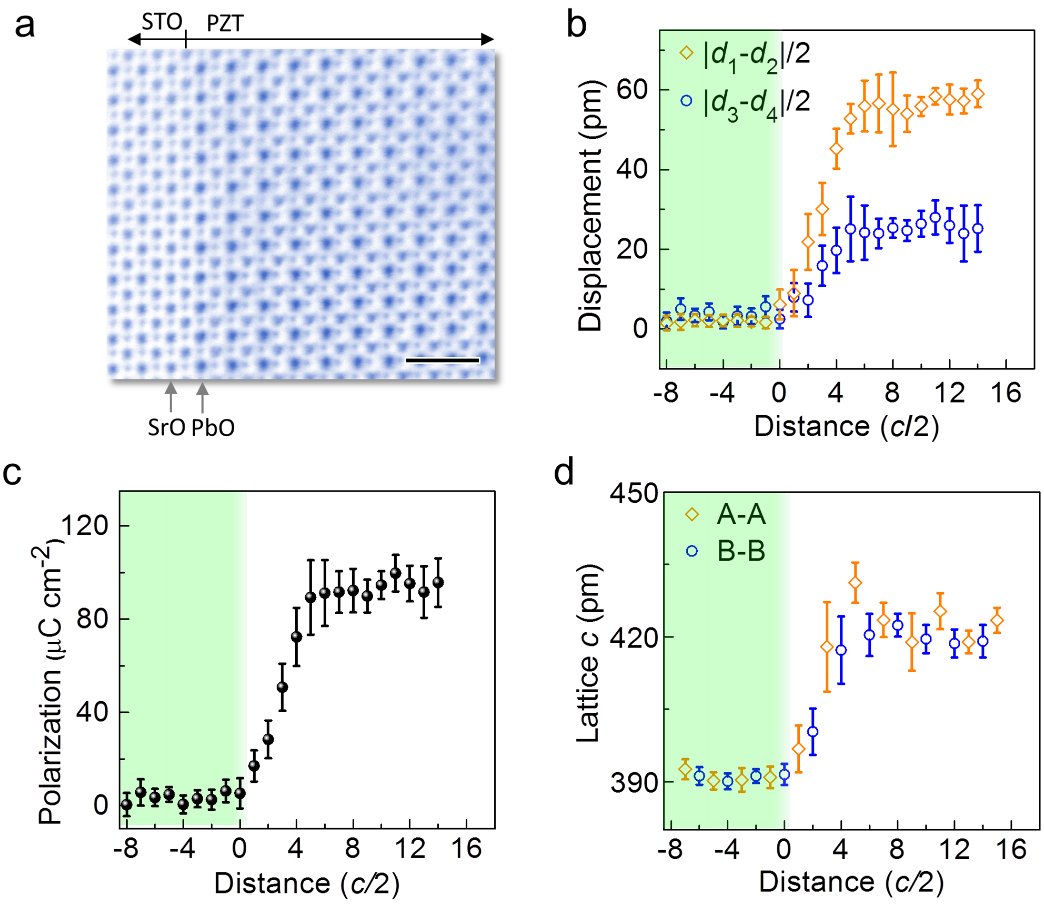

Supplementary Figure 1 | A region with thickness about 18–20 unit cells. (a) The annular bright filed (ABF) image. Scale bar: 1 nm. (b) Calculated displacements of A (Sr or Pb) respective to O and B (Ti or Zr/Ti) respective to O. The error bar is the standard deviation (s.d.). (c) Calculated total polarization. The error bar is the s.d. (d) Calculated lattice *c* from cation sublattice. The error bar is the s.d. These measured structural parameters of PZT thin film are basically in agreement with the previous X-ray study of $PbZr_{0.2}Ti_{0.8}O_3$ powder [1].

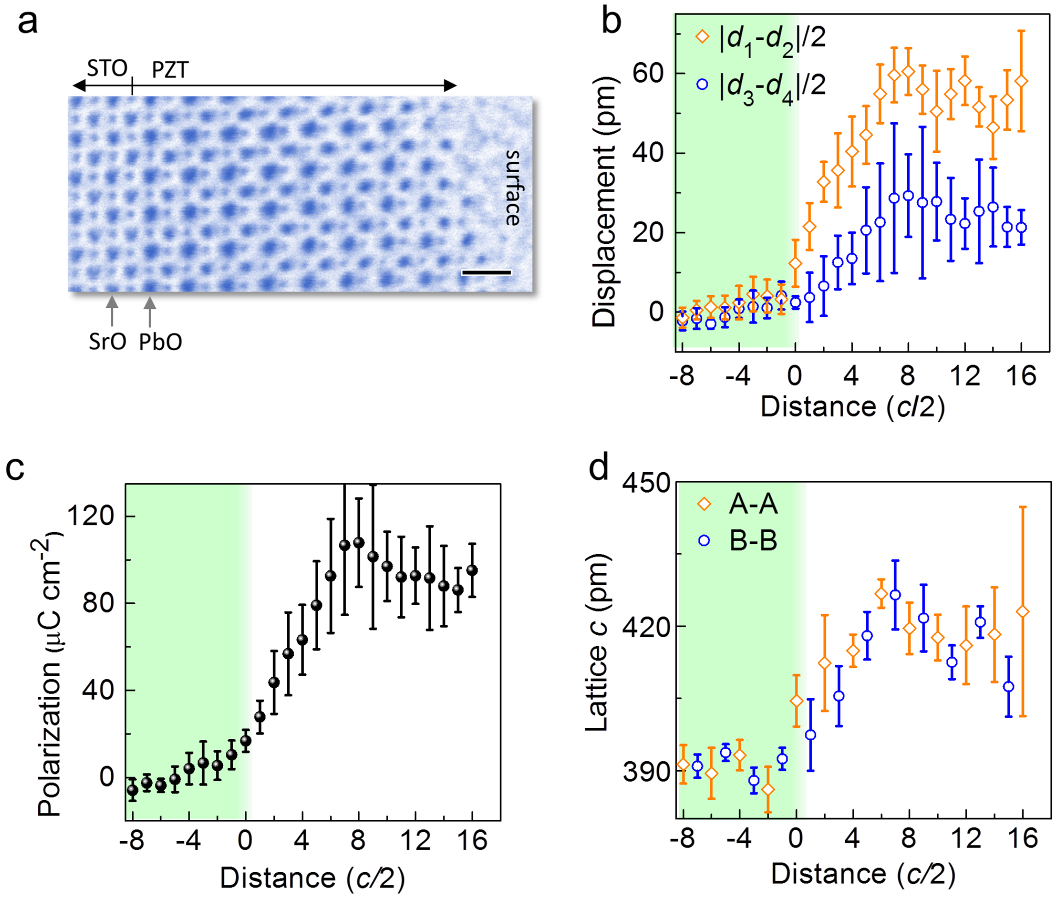

Supplementary Figure 2 | A region with thickness of 8.5 unit cells. (a) The ABF image. Scale bar: 0.5 nm. (b) Calculated displacements of A (Sr or Pb) respective to O and B (Ti or Zr/Ti) respective to O. The error bar is the s.d. (c) Calculated total polarization. The error bar is the s.d. (d) Calculated lattice *c* from cation sublattice. The error bar is the s.d.

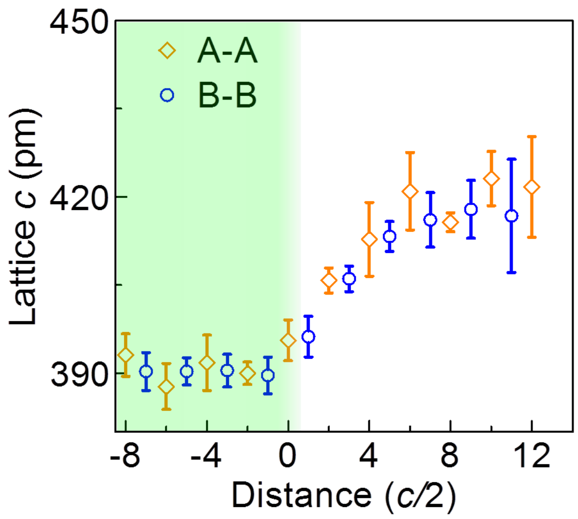

Supplementary Figure 3 | A region with thickness of 6.5 unit cells. Calculated lattice *c* from cation sublattice. The error bar is the s.d.

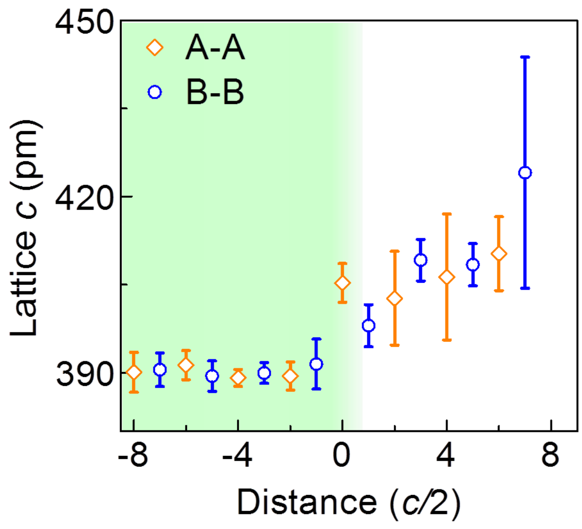

Supplementary Figure 4 | A region with thickness of 4 unit cells. Calculated lattice *c* from cation sublattice. The error bar is the s.d.

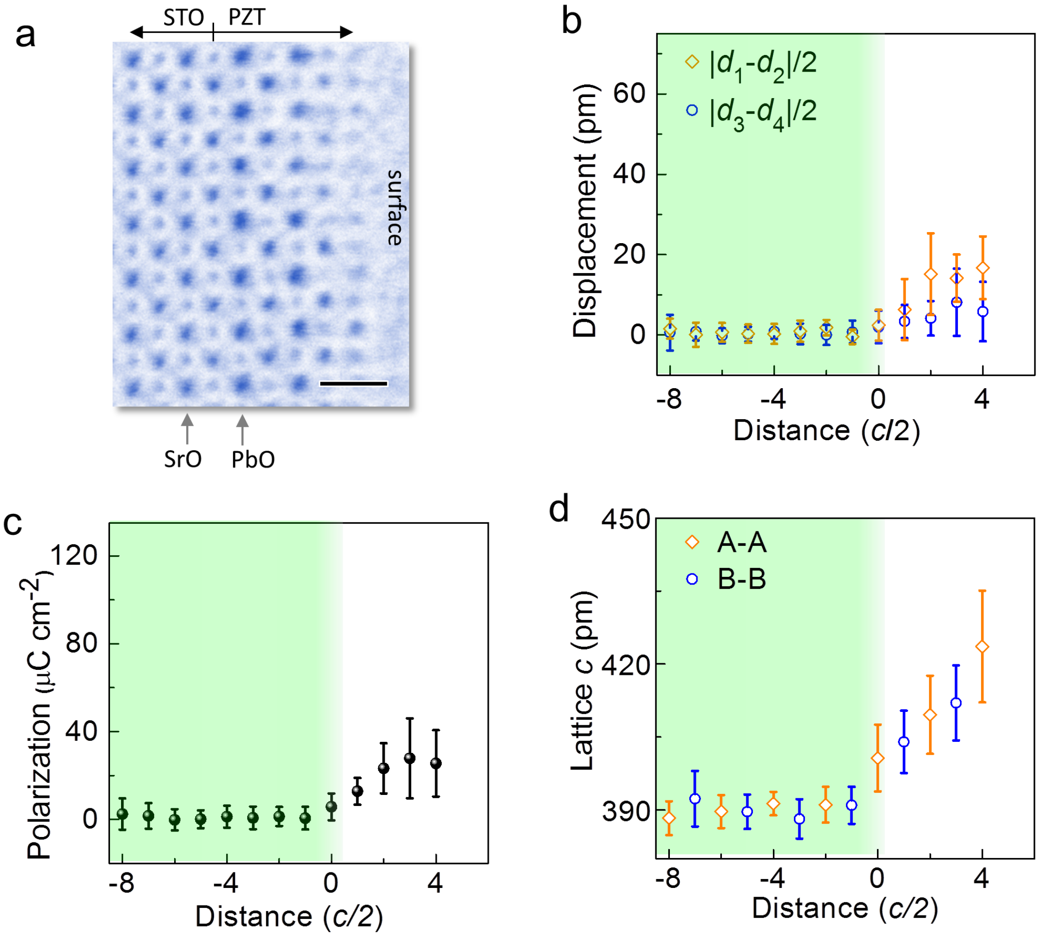

Supplementary Figure 5 | A region with thickness of 2.5 unit cells. (a) The ABF image. Scale bar: 0.5 nm. (b) Calculated displacements of A (Sr or Pb) respective to O and B (Ti or Zr/Ti) respective to O. The error bar is the s.d. (c) Calculated total polarization. The error bar is the s.d. (d) Calculated lattice *c* from cation sublattice. The error bar is the s.d.

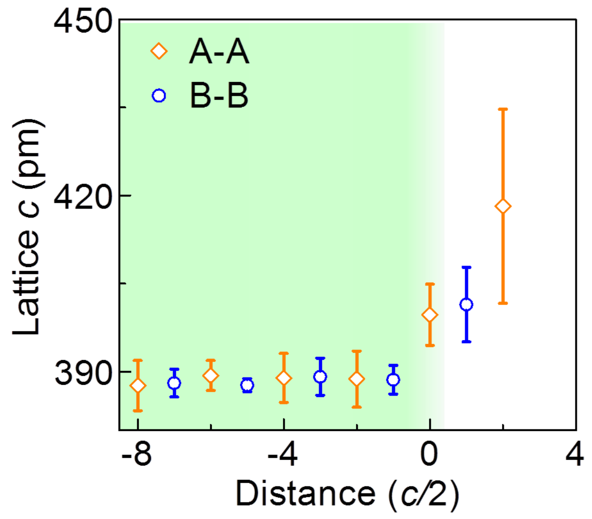

Supplementary Figure 6 | A region with thickness of 1.5 unit cells. Calculated lattice c from cation sublattice. The error bar is the s.d.

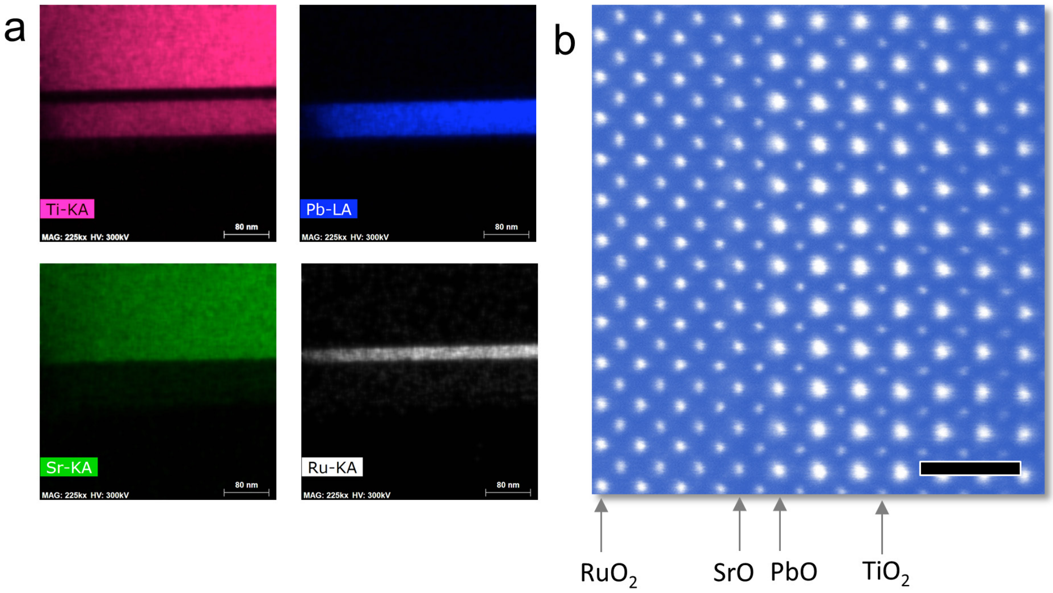

Supplementary Figure 7 | $PbZr_{0.2}Ti_{0.8}O_3$ thin film on $SrTiO_3$ substrate with $SrRuO_3$ bottom electrode. (a) Elemental mapping. (b) High angle annular dark field (HAADF) image. Scale bar: 1 nm.

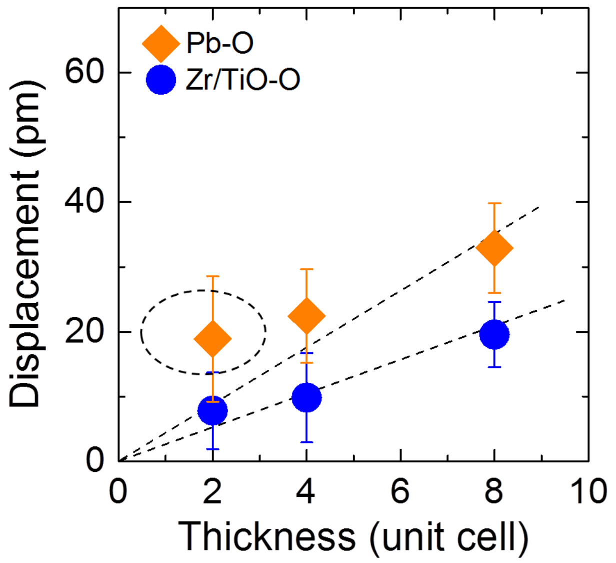

Supplementary Figure 8 | Robust Pb-O bond in the ultrathin $PbZr_{0.2}Ti_{0.8}O_3$ film on $SrRuO_3/SrTiO_3$. Displacements of Pb respective to O and Zr/Ti respective to O are plotted as a function of the thickness. The error bars are the s.d.

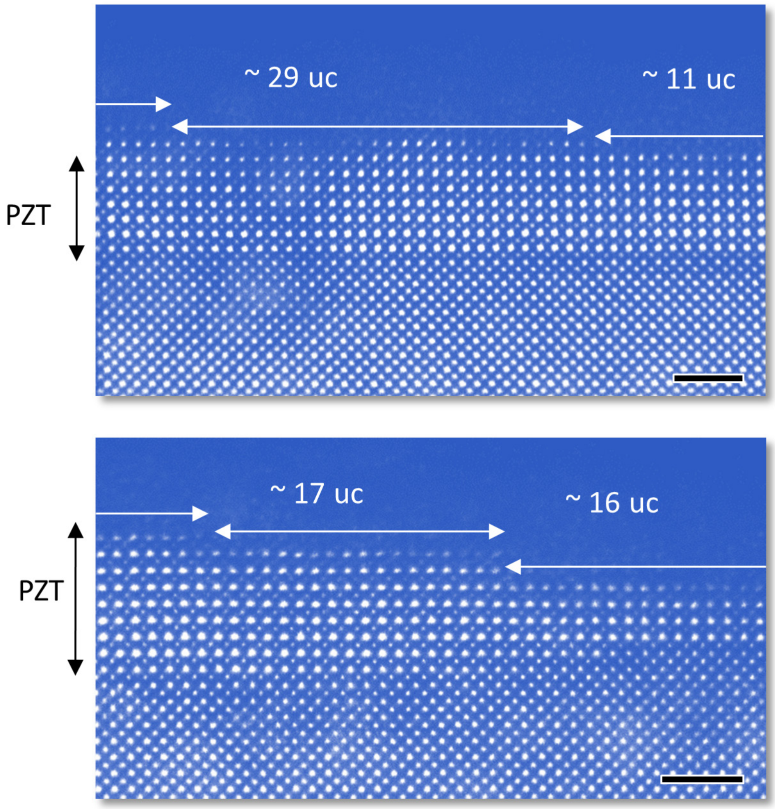

Supplementary Figure 9 | The atomic terrace in the thin regions in $PbZr_{0.2}Ti_{0.8}O_3$ films. The typical width of a terrace is from 10 to 30 unit cells. Scale bar: 2 nm.

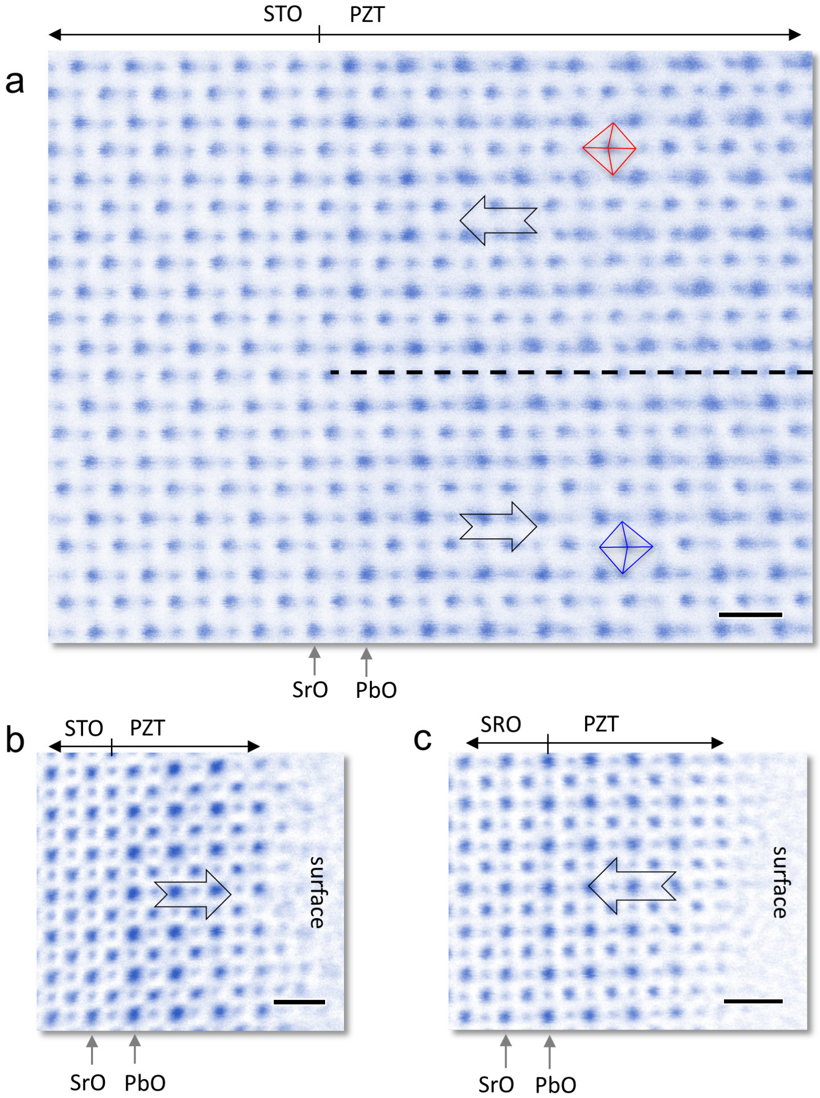

Supplementary Figure 10 | The switchable nature of polarization. (a) In a relative thick region, both upward and downward domains are present in $PbZr_{0.2}Ti_{0.8}O_3$ films on $SrTiO_3$ substrate, indicating the switchable nature. The domain wall position is highlighted by the dashed line. Scale bar: 0.5 nm. (b) Upward polarization in $PbZr_{0.2}Ti_{0.8}O_3/SrTiO_3$ heterostructure. Scale bar: 0.5 nm. (c) Downward polarization in $PbZr_{0.2}Ti_{0.8}O_3/SrRuO_3/SrTiO_3$ heterostructure. Scale bar: 0.5 nm.

Supplementary Reference

1 Joseph, J. *et al.* Structural investigations on $Pb(Zr_xT_{1-x})O_3$ solid solutions using the X-ray Rietveld method. *J. Mater. Sci.* **35**, 1571-1575 (2000).
